# Connecting Cryptococcal Meningitis and Gut Microbiome

**DOI:** 10.3390/ijms241713515

**Published:** 2023-08-31

**Authors:** Yuanyuan Ma, Liang Yang, Mengna Jiang, Xinyuan Zhao, Peng Xue

**Affiliations:** 1 Nantong Key Laboratory of Environmental Toxicology, Department of Occupational Medicine and Environmental Toxicology, School of Public Health, Nantong University, Nantong 226019, China; myycsd@hotmail.com (Y.M.); 17851790126@163.com (M.J.); 2 School of Medicine, Southern University of Science and Technology, Shenzhen 518055, China; yangl@sustech.edu.cn

**Keywords:** cryptococcal meningitis, melanin, catecholamines, nutritional signals, microbiota–gut–brain axis

## Abstract

Fungal pathogens of the *Cryptococcus neoformans* species complex (*C. neoformans* SC) are a major cause of fungal meningitis in immunocompromised individuals. As with other melanotic microorganisms associated with human diseases, the cell-wall-associated melanin of *C. neoformans* SC is a major virulence factor that contributes to its ability to evade host immune responses. The levels of melanin substrate and the regulation of melanin formation could be influenced by the microbiota–gut–brain axis. Moreover, recent studies show that *C. neoformans* infections cause dysbiosis in the human gut microbiome. In this review, we discuss the potential association between cryptococcal meningitis and the gut microbiome. Additionally, the significant potential of targeting the gut microbiome in the diagnosis and treatment of this debilitating disease is emphasized.

## 1. Introduction

Strains belonging to the *Cryptococcus neoformans* species complex (*C. neoformans* SC) are human pathogenic fungus, classified within the phylum Basidiomycota [1,2]. Although it possesses a clearly defined bipolar mating system, consisting of two distinct mating types, MATa and MATα [3,4], it is also capable of undergoing self-fertilization within cells of the same mating type [5,6]. *C. neoformans* SC fungi are broadly distributed in the environment, particularly in bird guano, soil, and trees [7]. *C. neoformans* SC fungi are especially dangerous for immunocompromised hosts including the HIV/AIDS population and organ transplant recipients due to their propensity to cause meningoencephalitis [8,9,10,11,12,13]. For example, *C. neoformans* SC fungi cause around 223,100 new cases and 181,000 deaths annually among individuals with HIV/AIDS [14,15]. Importantly, these species are classified as one of the four species in the highest priority group of fungal pathogens affecting humans, as identified by the World Health Organization (WHO). *C. neoformans* infections commonly occur through the inhalation of fungal spores or desiccated yeast cells in the environment [16,17], as well as through colonization of skin wounds [18]. These species are able to overcome host defenses and enter the bloodstream, where they can disseminate to various organs including the central nervous system (CNS), causing meningitis [16,19]. The mortality rate of cryptococcal meningitis is high, particularly in developing countries where access to effective treatments is limited. Despite all the efforts being made to create an effective vaccine, there is currently no reliable method for preventing infection. Even the most advanced treatment methods, which use a combination of antifungal agents, can only bring down the 10-week mortality rate to 24% [20]. Treatment of cryptococcal infections typically involves a combination of antifungal drugs, such as amphotericin B and flucytosine [8,20,21,22]. Successful phase III trial results on the combination therapy of amphotericin B and flucytosine have led to the WHO updating its treatment guidelines for cryptococcal disease in HIV-positive patients [22,23]. However, the emergence of drug-resistant strains of *C. neoformans* SC is a growing concern, highlighting the need for continued research into new therapies and preventative measures [20,24,25,26,27,28]. Importantly, recent studies have provided evidence that certain strains of probiotics and symbiotic bacteria residing in the intestines possess the ability to inhibit fungal invasion and colonization. Intestinal bacteria have emerged as a potent weapon in the treatment of fungal infections, including cryptococcal infections [29,30].

The diagnosis of cryptococcal meningitis typically involves a comprehensive evaluation of the clinical symptoms and signs, as well as the use of various diagnostic methods [21,31,32,33]. These methods involve evaluating the patient’s medical history and conducting a physical examination to identify symptoms associated with cryptococcal meningitis. Additionally, a lumbar puncture is commonly performed to collect and analyze cerebrospinal fluid (CSF) for the presence of *Cryptococcus* spp. Laboratory tests, including India ink staining of CSF, culture of *Cryptococcus* spp. by PCR, or antigen testing, are also employed to confirm the presence of cryptococcal infections. Various neuroimaging techniques, such as computed tomography (CT) imaging and magnetic resonance imaging (MRI), can be used to examine the brain for signs of meningitis or other abnormalities. The integration of these methods is essential for an accurate diagnosis and to rule out other potential causes. Research suggests that the composition of the gut microbiome may have a potential role in the diagnosis of cryptococcal meningitis. Studies have shown that alterations in the gut microbiome may be associated with fungal infections, including *Cryptococcus* spp. [34]. By analyzing the microbial communities in the gut, it may be possible to identify specific changes or markers that can aid in the diagnosis of cryptococcal meningitis. However, further research is needed to validate and establish the use of gut microbiome testing as a diagnostic tool for cryptococcal meningitis.

*C. neoformans* SC fungi have the ability to impair the host’s accurate identification of the fungal antigen and evade the immune response orchestrated by host phagocytes (i.e., macrophages or dendritic cells), T and B lymphocytes, innate lymphoid cells, and peripheral cytokines [35,36]. Moreover, *C. neoformans* SC fungi utilize melanin production to evade host immunity and enhance its infectivity in the host. The *C. neoformans* melanin in the cell wall, the major virulence factor, has various functions such as protecting against oxidative stress, reducing the efficiency of antifungal drugs, and affecting interactions with phagocytic cells [16,37,38]. Researchers believe that melanin could be a potential target for the development of agents against infections caused by the cryptococcal species [20,39]. One distinguishing characteristic of the *C. neoformans* laccase enzyme is its inability to generate melanin pigments from endogenously synthesized compounds, such as tyrosine. The production of melanin by using tyrosine is typical of these organisms [40]. In the brain tissue, the fungi of *C. neoformans* SC produce melanin pigments by oxidizing exogenous catecholamines (i.e., dopamine, norepinephrine, and epinephrine) through the laccase enzymes (Lac1 and Lac2) [41,42,43]. The neurotropism of *C. neoformans* SC is notably linked to the presence of catecholamines in the brain, along with low concentrations of glucose in the CNS. This is because under these conditions, laccase production is overregulated [42,44,45]. Recent research suggests that the microbiota–gut–brain axis (MGBA) can regulate the levels of *C. neoformans* melanin substrates in the brain [46]. MGBA can also potentially influence the host’s nutritional signals, such as glucose [47], that regulate melanin formation [45]. *C. neoformans’* melanization and the MGBA may be crucial for the development of effective antifungal agents for cryptococcal infections, including meningitis. In this review, we provide an overview of the existing knowledge regarding the possible links between cryptococcal meningitis and the gut microbiome. By gaining a deeper understanding of the connection, we may be able to develop more effective strategies for tests or treatments for cryptococcal meningitis.

## 2. *C. neoformans* Infections Cause Gut Microbiome Disruption

Recent advancements in our understanding and analysis of the gut microbiota have unveiled the significant impact of alterations on human health. The gut microbiota, an intricate and diverse microbial ecosystem, plays a vital role in various aspects of the host’s physiology. It actively participates in the host’s immune response, influences metabolism, facilitates biosynthesis, and defends against the pathogenic yeast infections [48,49,50,51]. In instances, the composition of the gut bacterial microbiota has been found to influence the generation of the pulmonary IL-17 response during the opportunistic human fungal pathogen *Aspergillus fumigatus* infections in mice. Mice with a specific composition of gut bacteria exhibit a more robust IL-17 response, which is crucial for combating fungal infections [52]. Furthermore, systemic infection with the invasive fungal pathogen *Candida albicans* has been shown to negatively impact the composition and diversity of the gut microbiota. This disturbance in microbial diversity can disrupt the delicate equilibrium within the gut ecosystem, potentially affecting overall gut health and immune function [53]. Similarly, studies have demonstrated the critical role of the microbiota in the host’s defense against *Cryptoccocus gattii* infections. Germ-free mice, lacking a microbiota, exhibited heightened susceptibility to *C. gattii* infections, resulting in lower survival rates and increased fungal burden in the brain and lungs. Furthermore, they displayed reduced levels of key immune factors such as IL-17, interleukin (IL)-1β, and interferon-γ, as well as decreased phosphorylation of the nuclear factor κB p65, compared to their wildtype counterparts [54]. In summary, the commensal microbiota plays a significant role in modulating immune responses during invasive fungal infections. Manipulating its composition holds promise as a potential therapeutic approach.

**Figure 1:**
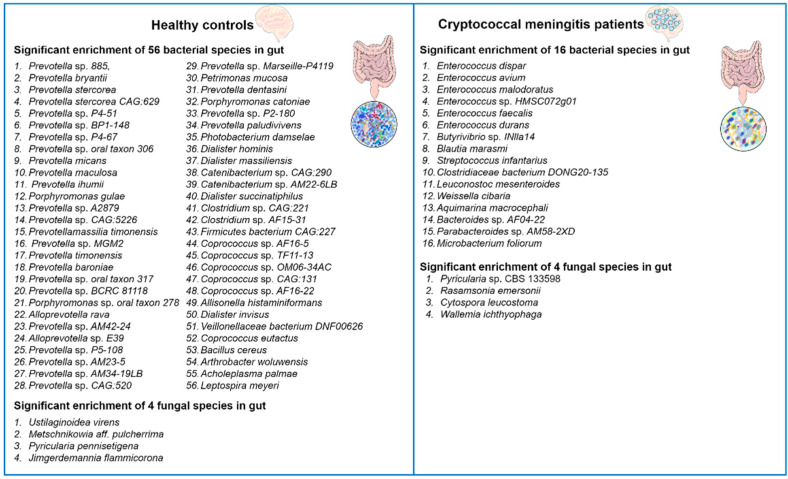
Diversity alterations in bacterial and fungal microbiota in the gut of cryptococcal meningitis patients. Compared to healthy controls, patients with cryptococcal meningitis exhibited distinctive compositions of both bacterial and fungal microbiota. A total of 72 bacterial species and 8 fungal species were found to be differentially abundant between the two groups [34].

Recently, Li and colleagues reported that *C. neoformans* infections induced alterations in the gut microbiota of humans [34]. The authors performed alpha and beta diversity analyses to compare the gut microbiota of patients with cryptococcal meningitis to healthy controls. The results showed that patients with cryptococcal meningitis had significantly lower alpha diversity compared to healthy controls, indicating gut dysbiosis. In total, they identified 72 differentially abundant bacterial and 8 differentially abundant fungal species between these two groups (Figure 1). For example, the patients with cryptococcal meningitis had a higher abundance of the bacteria *Enterococcus avium*, *Microbacterium foliorum*, and *Bacteroides* spp. and of the fungi *Pyricularia* spp., *Cytospora leucostoma,* and *Wallemia ichthyophaga*, and a lower abundance of the bacteria *Prevotella* spp., *Coprococcus* spp., and *Arthrobacter woluwensis* and of the fungi *Jimgerdemannia flammicorona*, *Metschnikowia aff. Pulcherrima*, and *Pyricularia pennisetigena* as compared to the healthy controls. Interestingly, antifungal treatment had only minor effects on the gut microbiota composition, suggesting that *C. neoformans* infections causes long-lasting gut microbiota dysbiosis in the cryptococcal meningitis patients. To further explore the potential correlations between bacteria, fungi, and clinical indicators of cryptococcal meningitis, the authors performed correlation analyses. Several bacterial and fungal taxa were found to be positively or negatively correlated with the disease-related symptoms, such as visual disorders and auditory symptoms. Overall, Li et al.’s study [34] provides valuable insights into the significant impact of *C. neoformans* infections on the gut microbiota and the potential associations between cryptococcal meningitis and gut microbiome disruption. 

## 3. *C. neoformans’* Melanization in Human Brain Tissue

**Figure 2:**
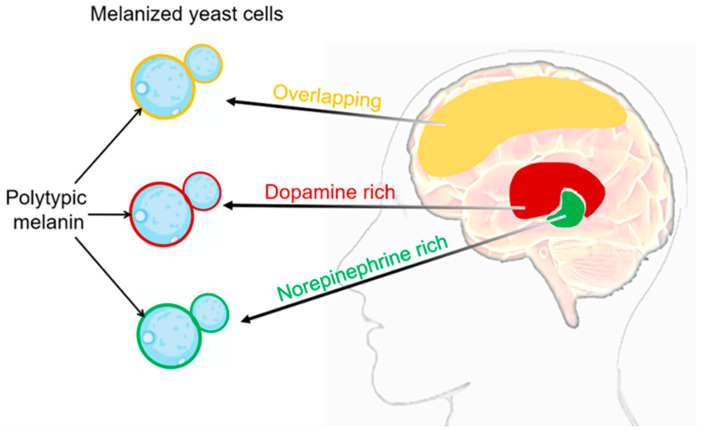
*C. neoformans’* melanization in different brain tissues. The composition of melanin generated during infection exhibits variations contingent upon the catecholamine composition of the tissue. It is highly probable that the in vivo synthesis of the melanin pigment arises from the polymerization of a diverse assortment of precursor compounds [37,41]. Catecholamine distribution in the brain: red domain represents dopamine rich, green domain represents norepinephrine rich, yellow domain represents overlapping between dopamine and norepinephrine. In the cell wall of *C. neoformans* SC, the respective types of melanin produced in different brain regions can be visualized as red, green, and yellow.

For *C. neoformans* SC fungi, the cell surface features that contribute to pathogenesis include the deposition of melanin in the cell wall [55,56]. The melanin deposition in *C. neoformans* SC is determined by the composition and flexibility of its cell wall [57]. Melanin deposition within the cell wall offers several advantages to the pathogen [58]. Firstly, melanin acts as a protective barrier against host immune responses, including phagocytosis by immune cells. Melanin has been shown to inhibit the production of reactive oxygen species and diminish the activity of antifungal agents, thus increasing the resistance of *C. neoformans* SC to host defenses. Moreover, melanin has been implicated in the dissemination of *C. neoformans* SC within the host. Melanized fungal cells have been detected in various organs, including the brain and lungs, indicating that melanization plays a role in the invasion and establishment of infections in different tissues [59]. The primary indication of *C. neoformans’* melanization during infection came from the identification of acid-resistant melanin ghost particles. These particles were isolated from infected animal and human tissue, as well as from cells cultivated on agar plates with tissue homogenate [60,61]. The nervous system of mammals serves as an abundant source of precursors in the form of catecholamines, which are nitrogen-containing diphenolic compounds. These catecholamines include neurotransmitters like dopamine, epinephrine, and norepinephrine [62,63]. *C. neoformans* SC fungi produce melanin by catalyzing the oxidation of exogenous catecholamine substrates using laccase enzymes [64,65,66]. Melanized *C. neoformans* cells were detected in brain tissue samples from patients with cryptococcal meningitis [60]. The melanin synthesized by *C. neoformans* SC fungi in brain tissue may vary in different anatomical regions due to its ability to incorporate multiple catecholamines simultaneously (Figure 2). This is because the relative proportions of these neurotransmitters can differ significantly from one area of the brain to another [62,63]. The chemical structure of the substrate added to the media dictates the variability of the synthesized pigment type. It is crucial to note that Baker et al. made an important observation regarding the ability of *C. neoformans* SC to exploit the human brain catecholamine mixture (0.6 mM dopamine, 0.33 mM norepinephrine, and 0.07 mM epinephrine) to produce polytypic melanin. This melanin provides protection against UV light and oxidants. Furthermore, they observed that the amount of melanin during the infection was influenced by the specific catecholamines present in the tissue. They also explored the possibility of utilizing dopamine as a precursor chemical to enable the fungi to produce a pigment with similar properties [41]. This finding highlights the adaptability and resourcefulness of the *C. neoformans* SC in utilizing specific components found in the host environment to promote its survival and pathogenesis. Interestingly, *C. neoformans* infections show a notable concentration in the basal ganglia region of the brain, where dopamine levels are highest [67]. This finding raises intriguing possibilities, such as the potential resistance of melanized cells to immune clearance mechanisms. Additionally, this preference for the basal ganglia region aligns with the hypothesis that the melanin produced by *C. neoformans* SC strains in the brain is primarily derived from dopamine. Taken together, the detection of melanized fungal cells in human brain tissue samples highlights the ability of *C. neoformans* SC to undergo melanization within the brain. The presence of multiple catecholamines within the human brain provides substrates for polytypic melanin synthesis (Figure 2), thereby augmenting the virulence and survivability of the fungus. The extent of melanization varied among different brain regions, with higher levels observed in the basal ganglia and thalamus. This suggests that melanization may play a role in the localization and dissemination of *C. neoformans* SC within the brain.

## 4. The Possible Impact of the Gut Microbiome on *C. neoformans’* Melanization in Brain

### 4.1. Gut Microbiome Influences the Levels of Melanin Substrates

**Figure 3:**
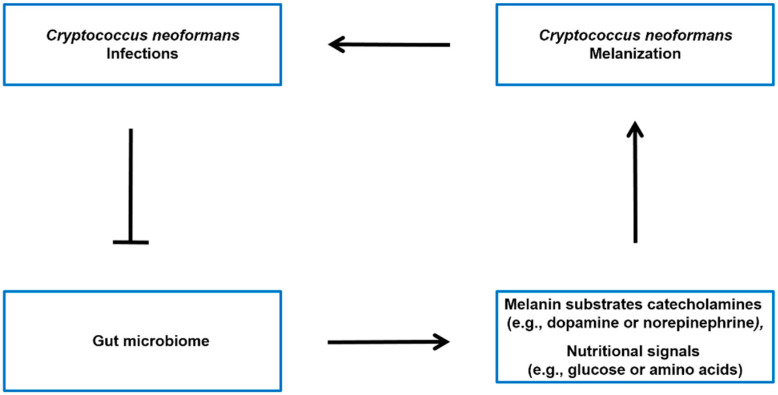
Possible model of the interconnection between *C. neoformans* infections, the gut microbiome, and host molecules throughout the body. *C. neoformans* infections have been associated with alterations in the gut microbiome [34]. The microbiota–gut–brain axis may regulate the levels of melanin substrates catecholamines (e.g., dopamine or norepinephrine) [46,71,73], as well as the nutritional signals (e.g., glucose or amino acids) [47] that modulate the melanin formation of *C. neoformans* SC in the brain.

The gut–brain axis is a bidirectional communication system connecting the gut microbiome and the central nervous system [68,69]. It is widely recognized that the gut microbiome has the ability to impact various aspects of brain function, such as mood, behavior, and cognition [70,71]. This is believed to happen through different pathways, such as the production of neurotransmitters and other signaling molecules that can influence the functioning of the central nervous system [70,72]. While there is no direct evidence linking the gut–brain axis to the synthesis of *C. neoformans* melanin, evidence suggests that the gut microbiome has the potential to influence levels of catecholamines (Figure 3), such as dopamine and norepinephrine, which are the melanin substrate for *C. neoformans* SC strains in brain tissue. For instance, recent studies have indicated that the level of striatal dopamine is regulated by the colonization of intestinal microbes. Metabolites produced by gut bacteria are absorbed into the bloodstream and can cross the blood–brain barrier, where they have an impact on the dopamine system in the brain. Germ-free mice, which were colonized with a modified strain of *Escherichia coli* containing genes responsible for fatty acid amides (FAAs) biosynthesis, exhibited improved running performance on both exercise wheels and treadmills compared to the control group. Furthermore, the activation of the endocannabinoid receptor CB1, which is expressed in neurons that also express vanilloid receptor TRPV1, by FAAs led to an increase in sensory neuron activity, the release of dopamine in the striatum, and an overall enhancement in exercise performance [46]. Additionally, germ-free mice have higher turnover rates of catecholamines, dopamine, and norepinephrine in the brain [71]. The levels of norepinephrine in the hippocampus were significantly lower in mice with antibiotics-induced depression compared to normal mice [73]. In conclusion, the gut microbiome has the potential to influence *C. neoformans* melanin substrate levels. 

### 4.2. Gut Microbiome Impact on Nutritional Signals That Regulate Melanization

The ability to quickly adapt to fluctuating external conditions is vital for the survival and propagation of microorganisms. This is especially significant for pathogenic microbes as they need to navigate the shift from the environment to the host milieu and initiate an appropriate response to establish an infection. Hosts pose challenging conditions, such as varying nutrient availability, oxygen levels, pH levels, and temperature, along with the potential threats posed by the host immune response [74,75]. It is interesting to note that the mechanisms of adapting to nutrient availability not only facilitate the proliferation of microorganisms but also play a role in regulating their virulence [76]. Glucose levels, for example, have been found to be a key factor in promoting melanin synthesis in *C. neoformans* fungi [45]. When the fungus is starved of glucose, it responds by increasing its melanin production. Signal transduction pathways are crucial in facilitating microbial adaptation. The cAMP-PKA nutrient sensing pathway has also been discovered to play a crucial role in regulating melanin synthesis in *C. neoformans* SC fungi. A glucose and methionine mixture has been shown to increase cAMP accumulation and PKA activation, leading to increased melanin synthesis [77,78]. The gut microbiome can regulate the availability of nutrients such as glucose and amino acids for *C. neoformans* SC fungi [47]. Imbalances or alterations in the gut microbiome composition may therefore affect the levels of these nutritional signals, potentially influencing melanization processes in the body (Figure 3). 

## 5. Conclusions

The current understanding of the relationship between cryptococcal meningitis and the gut microbiome is limited, but recent studies have shed light on potential connections. While no direct link has been established, evidence suggests that the presence of *C. neoformans* infections can have a significant impact on the composition and function of the gut microbiota. Studies have shown that the gut microbiome may play a role in influencing *C. neoformans* melanin formation. Melanin is a key component of the cryptococcal cell wall and is known to contribute to the pathogenicity of the fungus. It has been proposed that certain gut microbial species may modulate the availability of melanin precursors or provide metabolic support for melanin synthesis in *C. neoformans* SC fungi. This hypothesis opens up new avenues for research into the specific microbial species and their potential involvement in melanin production. While the correlations between cryptococcal meningitis and the gut microbiome are still being actively investigated, it is clear that further studies are needed to fully understand the underlying mechanisms of this interaction. Future research should focus on elucidating the specific microbial species that are associated with *C. neoformans* infections and determining their functional roles in modulating disease progression. The potential implications of this research are significant. By gaining a better understanding of the relationship between cryptococcal meningitis and the gut microbiome, novel strategies for the prevention and treatment of *C. neoformans* infections can be developed. Targeting the gut microbiome through probiotics, prebiotics, or other interventions may offer a new approach to enhance the host’s immune response against the fungus or interfere with its pathogenic mechanisms. Furthermore, studying the gut microbiome in the context of cryptococcal meningitis may also provide insights into the broader field of infectious diseases. The gut microbiota has been implicated in the regulation of immune responses and the maintenance of overall health. Therefore, understanding how alterations in the gut microbiome influence the development and progression of cryptococcal meningitis could have implications beyond this specific infection. While the exact connection between cryptococcal meningitis and the gut microbiome remains unclear, recent studies have highlighted the impact of *C. neoformans* infections on the gut microbiota. Further research is needed to explore the role of specific gut microbial species in modulating melanin formation and to unravel the underlying mechanisms of this connection. This area of research holds promise for the development of innovative approaches to prevent and treat *C. neoformans* infections by targeting the gut microbiome.

## Data Availability

Not applicable.
